# Peripartum cardiomyopathy: a KKH case series

**DOI:** 10.1186/cc9433

**Published:** 2011-03-11

**Authors:** MK Shah, S Leo, CE Ocampo, CF Yim, S Tagore

**Affiliations:** 1 Kandang Kerbau Women's and Children's Hospital, Singapore

## Introduction

The incidence, presentation and risk factors of peripartum cardiomyopathy in Singapore are not known.

## Methods

Seven patients' case notes were reviewed following IRB approval.

## Results

Incidence was 1:2,285 deliveries. Symptoms appeared 1 hour post-LSCS delivery intraoperatively to postpartum day 5, with diagnosis within a few days. Dyspnoea, desaturation, frusemide-induced diuresis, and CXR evidence of pulmonary congestion/oedema occurred in all. Troponin I (measured in 6/7 cases) and CKMB (measured in 5/7) were raised, and then (troponin I repeated in 4/6 and CKMB repeated in 3/5) showed a declining trend. BNP and CRP (measured in Case 6 only) were raised. 2D-ECHO showed worst LVEF 25 (19 to 35)%, median (range), at time of diagnosis, <25% (Cases 1 and 3), valvular abnormalities (4/7), LV diastolic dysfunction (2/7), two-chamber enlargement (3/7), one-chamber enlargement (1/7), and follow-up 2D-ECHO (done in 5/7) showed last LVEF 55 (35 to 65)%, median (range) (Cases 1 and 6, <45%), and valvular abnormalities (3/7). All were Asian (except for one German, typical of our hospital's ethnic mix), mean age was 29.7 years (with only one older: 38 years), mean parity was 1.67 (6/7), all had singleton pregnancy, mean BMI was 28.2 (6/7, one with BMI: 36.1), and preterm labour (3/7, two of which had failed tocolysis with oral adalat and i.v. salbutamol), prostin induction of labour (3/7), caesarean delivery (3/7), and postpartum haemorrhage (3/7) were also noted. They were all managed aggressively without delay. Treatment included oxygen therapy (all), intubation, sedation and ventilation (6/7), BiPAP (3/7), pleural drainage (2/7), frusemide, digoxin and ACE inhibitors (for example, perindopril, enalapril) (all), antibiotic(s) for pneumonia (for example, tazocin, coamoxiclav, ceftriaxone, clarithromycin, doxycycline, gentamicin, metronidazole) (6/7), anticoagulant/antiplatelet prophylaxis (for example, fraxiparine, clexane, aspirin, warfarin) (6/7), beta-blockers (for example, carvedilol, bisoprolol, labetalol) (5/7), other inotropes, namely dobutamine (2/7, in one patient with noradrenaline) and milrinone (1/7), and vasodilators, namely GTN and hydralazine (1/7). Total hospitalisation from time of diagnosis was 5 to 9 days. Following 4 (1 to 8) months, median (range), follow-up, 4/7 made full recovery, 1/7 partial recovery, 1/7 temporary recovery, and 1/7 defaulted. Case 2 resulted in a neonatal death.

## Conclusions

Possible risk factors are multiparity, preterm labour requiring tocolysis, prostin induction of labour, and postpartum haemorrhage.

